# Gene Expression Profiling Provides an Improved Characterization of *CD79B*-Mutated Diffuse Large B-Cell Lymphomas

**DOI:** 10.3390/jpm15110548

**Published:** 2025-11-10

**Authors:** Luis Grossmann, Wolfgang Jagla, Marcus Bettstetter, Simone Bertz, Stephan Schwarz-Furlan, Thomas Richter, Tobias Dechow, Thomas Decker, Martin Dreyling, Karl Sotlar, Harald Bartsch, Arndt Hartmann, Julius Honecker, Andreas Gaumann

**Affiliations:** 1 Institute of Pathology, Universitätsklinikum Erlangen, Friedrich-Alexander-Universität Erlangen-Nürnberg (FAU), Krankenhausstrasse 8-10, 91054 Erlangen, Germany; simone.bertz@uk-erlangen.de (S.B.); stephan.schwarz@pathologie-memmingen.de (S.S.-F.); arndt.hartmann@uk-erlangen.de (A.H.); 2 Institute of Pathology Kaufbeuren-Ravensburg, Julius-Probst-Strasse 4, 87600 Kaufbeuren, Germanyandreas.gaumann@pathologie-kaufbeuren.de (A.G.); 3 Foundation Medicine GmbH, Nonnenwald 2, 82377 Penzberg, Germany; 4 Teilgemeinschaftspraxis Molekularpathologie Südbayern, Giesinger Bahnhofplatz 2, 81539 Munich, Germany; 5 Institute of Pathology Rosenheim, Lilienweg 12, 83022 Rosenheim, Germany; 6 Medizinisches Versorgungszentrum für Hämatologie und Onkologie Ravensburg GmbH, Elisabethenstrasse 19, 88212 Ravensburg, Germany; dechow@onkonet.eu (T.D.); decker@onkonet.eu (T.D.); 7 Medical Clinic and Policlinic III, Ludwig-Maximilians-Universität München, Marchioninistrasse 15, 81377 Munich, Germany; martin.dreyling@med.uni-muenchen.de; 8 Institute of Pathology, Ludwig-Maximilians-Universität München, Thalkirchner Strasse 36, 80337 Munich, Germany; k.sotlar@salk.at; 9 Institute of Pathology, University Hospital Salzburg, PMU Salzburg, Müllner Hauptstraße 48, A-5020 Salzburg, Austria; 10 Gemeinschaftspraxis für Pathologie Traunstein, Cuno-Niggl-Strasse 3, 83278 Traunstein, Germany; h.bartsch@pathots.de

**Keywords:** molecular pathology, DLBCL, lymphoma, gene expression, mutations, prognosis, *CD79B*, *MYD88*, NF-κB pathway, *TP53*

## Abstract

**Background and Objectives**: Diffuse large B-cell lymphomas (DLBCLs) are heterogeneous neoplasms. *CD79B* and *MYD88* mutations are associated with the activated B-cell-like (ABC) subtype of DLBCL and often co-occur and lead to constitutive activation of the NF-κB pathway. Several different genetic classifications to date have recognized *CD79B*- and *MYD88*-mutated DLBCLs as a unique subtype with poor response to therapy and unfavorable survival. However, little is known about gene expression in DLBCLs with mutated *CD79B* (and *MYD88*) in comparison to their wild type counterparts. The objective of this study was to compare the gene expression in DLBCLs according to their *CD79B* mutational status. **Methods**: A total of 48 primary, treatment-naïve DLBCLs (*CD79B*-mutated: 35%/n = 17, *CD79B*-wild type: 65%/n = 31) were investigated using RNA expression profiling (770 genes), followed by immunohistochemical analysis of the up-regulated genes and survival analysis. **Results**: The gene expression analysis revealed that downstream of CD79B *CARD11* and the NF-κB targets *NFKBIZ*, *IL10*, *IL12A*, *PIM1* and *BCL2A1* were up-regulated in *CD79B*-mutated DLBCLs. The strongest up-regulation was detected for *ARNT2* and *WNT11*. Other up-regulated genes included the apoptosis-related *BID* and *granzyme B*, as well as genes of cell cycle regulation such as *RUNX1*, *RUNX1T1* and *RASGRF1*. Up-regulation was also found for *IL7*, *STAT3*, *MLLT4*, *CD14* and the *HSP90B1* subunit. *TP53* mutation showed an association with poorer overall survival in a secondary analysis, consistent with prior reports, while survival by *CD79B*/*MYD88* mutation status and the differentially expressed genes showed no significant differences in this cohort. **Conclusions**: In conclusion, the current study identified novel up-regulated genes in *CD79B*-mutated DLBCLs beyond NF-κB pathway signaling, which may contribute to a better definition of potential therapeutic targets and further improves the characterization of this distinct and aggressive DLBCL subgroup.

## 1. Introduction

Diffuse large B-cell lymphoma (DLBCL) is the most frequent lymphoma, accounting for 40% of cases [1,2]. This class of non-Hodgkin’s lymphomas presents with manifestations in both nodal and extranodal locations and is characterized by a high degree of molecular heterogeneity that remains incompletely understood [3,4,5,6]. Based on gene expression profiling, DLBCL can be subdivided into different prognostic subgroups: germinal center B-cell-like (GCB) DLBCL, activated B-cell-like (ABC) DLBCL and unclassified [2,3,7]. Since gene arrays are costly and not practical in clinical routine, DLBCLs are often subdivided into GCB and non-GCB lymphomas using the immunohistochemical Hans classifier [8,9]. In the ABC subtype, activating mutations in *CD79B* and *MYD88* are frequent and often occur concomitantly leading to constitutive activation of the NF-κB pathway [10,11,12,13]. NF-κB activation is considered a central mechanism in the pathogenesis of this lymphoma subtype and is associated with poor survival [10,11,14]. *CD79B* mutations are frequently located in the immunoreceptor tyrosine-based activation motif (ITAM) region in ABC DLBCLs, resulting in chronic activation of B-cell receptor (BCR) signaling [11]. In MYD88, an adaptor protein in interleukin-1 (IL1) and toll-like receptor signaling, the amino acid substitution L265P is predominantly found [10]. A high prevalence of *CD79B* and *MYD88* mutations is found in extranodal DLBCLs such as in the breast, central nervous system (CNS) and testes [12,15,16,17,18,19]. Genetic subtyping of DLBCL revealed that the co-occurrence of mutations in the *MYD88* and *CD79B* genes defines a unique molecular subtype of DLBCLs, mostly termed MCD [20,21,22,23,24]. The MCD subtype was associated with poor response to standard therapy and unfavorable survival [20,24]. *CD79B* mutation was identified as an unfavorable prognostic factor for DLBCL patient survival and may be an important biomarker for DLBCL disease progression [25].

However, little is known about the gene and protein expression of DLBCLs with *CD79B* (and *MYD88*) mutations in comparison to their wild type (wt) counterparts. The main objective of the current study was the comparison of gene expression of 770 cancer-associated genes in 48 primary, treatment-naïve DLBCLs according to their *CD79B* mutational status. For a subset of the up-regulated genes, a corresponding overexpression at the protein level was investigated by immunohistochemistry (IHC). Survival analysis was performed to assess the prognostic significance of clinical variables, mutation status and differentially expressed genes.

## 2. Materials and Methods

### 2.1. Patients

Formalin-fixed paraffin-embedded (FFPE) tissue specimens of 48 DLBCL patients were obtained from the Institutes of Pathology Kaufbeuren-Ravensburg, Rosenheim and Erlangen. All cases were newly diagnosed, primary DLBCL. The tumor tissue was collected at initial diagnosis prior to any systemic therapy. Thus, the profiled samples are treatment-naïve. Patient characteristics and first-line treatments are summarized in the supplement (Supplement Table S1). The study was conducted in accordance with the Declaration of Helsinki and was approved by the Ethics Committee of the medical faculty of the Friedrich-Alexander-Universität Erlangen-Nürnberg (Germany) (103_17 B). Informed consent was obtained from patients included in this study.

### 2.2. Mutation Detection Analyses

#### 2.2.1. Mutation Detection of CD79A/B and MYD88

Three representative 7 µm sections were cut from each FFPE tissue block, deparaffinized, rehydrated in a graded ethanol series and rinsed in distilled water. FFPE sections were manually micro- or macrodissected, depending on tumor cellularity and distribution, to enrich for tumor DNA and transferred into 2 mL Eppendorf tubes. Samples were digested with Proteinase K (Roche Diagnostics GmbH, Mannheim, Germany) overnight at 56 °C on a shaking device. DNA was then purified using a column-based extraction kit (Quiagen GmbH, Hilden, Germany) and stored at 4 °C until further analysis. All tumor DNA samples were screened for mutations in the ITAM region of *CD79A* and *CD79B* as well as for the point mutation L265P of *MYD88* by real-time polymerase chain reaction (real-time PCR) combined with high-resolution melting analysis (HRMA). Tumor samples with a melting curve deviating from the wild type control were further analyzed by bidirectional Sanger sequencing to verify and characterize the mutations.

#### 2.2.2. Mutation Detection of TP53

Sample DNA was amplified with target specific primers, followed by barcode incorporation to generate barcoded libraries covering *TP53*. PCR was performed using the FastStart^TM^ High Fidelity PCR-System according to the manufacturer’s instructions (Roche Diagnostics GmbH, Germany). Libraries were prepared using the Ion PGM^TM^ Hi-Q^TM^ View Chef Kit on the Ion Chef Instrument (Thermo Fisher Scientific Inc., Waltham, MA, USA). Sequencing of multiplexed templates was performed on the Ion PGM^TM^ System using the Ion PGM^TM^ Hi-Q^TM^ View Sequencing Kit on Ion 314/316 chips following the manufacturer’s instructions (Thermo Fisher Scientific Inc., USA). Data analysis was performed using SeqNext (JSI medical systems GmbH, Ettenheim, Germany).

### 2.3. Multi-Gene Expression Analysis

At least three representative 7 µm sections were cut from the respective FFPE tissues and deparaffinized as mentioned. Following micro- or macrodissection based on tumor content, the samples were digested with Proteinase K (Roche Diagnostics GmbH, Germany) overnight at 56 °C. RNA was extracted using the RNeasy Kit according to the manufacturer’s protocol (Quiagen GmbH, Germany). DNA was digested with DNase I and after several washing steps RNA was eluted. RNA yield and purity were measured using a spectrophotometer. The RNA samples were stored at −80 °C until further analysis. Gene expression analysis was performed using the nCounter^®^ PanCancer Pathways Panel according to the manufacturer’s instructions (NanoString Technologies Inc., Seattle, WA, USA). The panel includes 770 genes from 13 canonical pathways and selected reference genes. The input per sample was 100 ng RNA. The nCounter^®^ Analysis System (NanoString Technologies Inc., USA) generated the raw RCC files. These files were analyzed using the nSolver^TM^ Analysis Software 2.5 with the Advanced Analysis Module (NanoString Technologies Inc., USA). Three normal lymph nodes were used as baseline controls.

### 2.4. TMA and Immunohistochemistry

A tissue microarray (TMA) was constructed using 2 mm tissue cores embedded in three comparable tissue blocks. Several 2 µm sections were cut from at least two different TMA blocks and stained with hematoxylin and eosin (H&E) for morphological evaluation. Subsequent sections were deparaffinized after drying and rehydrated in a graded ethanol series. Antigen retrieval was performed by heat treatment. After the washing steps, primary antibodies were applied at the respective dilutions (Supplement Table S2). After washing, antibody binding was visualized with the ZytoChem Plus (AP) Polymer Bulk Kit for 30 min at room temperature (RT), employing Permanent AP Red (both from Zytomed Systems GmbH, Berlin, Germany) as the chromogenic substrate. Tissue sections were analyzed using an Axio Imager.A2 light microscope (Carl Zeiss AG, Oberkochen, Germany) and a MIRAX DESK slide scanner (Carl Zeiss AG, Germany) with a Pannoramic Scanner 1.22 and CaseViewer 2.4 software (3DHISTECH, Budapest, Hungary). Staining results were evaluated manually using a semi-quantitative scoring method based on the percentage of positive cells (0–100%) and the staining intensity, which was assessed using four levels: 0 = no immunoreactivity (IR); 1 = weak IR; 2 = medium IR; 3 = strong IR. For each sample, the staining intensities were multiplied by the respective percentage of positive tumor cells, resulting in an H-score ranging from 0 to 300. Protein expression was classified as negative for H-scores < 80 and positive for H-scores ≥ 80. The investigation of a corresponding protein expression for the detected up-regulated genes from the RNA expression analysis in the *CD79B*-mutated DLBCLs comprised the immunohistochemical analysis of granzyme B, PIM1, IL10, IL7, HSP90 and STAT3 in the lymphoma TMAs. NF-κB and p53 were also stained. Given the finite archival tissue, IHC analyses were pre-specified as exploratory and limited to a subset of up-regulated genes.

### 2.5. Survival Analysis

The survival analyses included univariate and multivariate examinations. The prognostic significance of clinical variables, mutation analysis results and gene expression analysis results were assessed separately. Both relapse-free survival (RFS) and overall survival (OS) were investigated using the Kaplan–Meier method (product-limit procedure), with the date of histological diagnosis as the starting point. Overall survival was defined as time to death from any cause, with censoring at last follow-up. Relapse-free survival was defined as time to first documented relapse, with censoring at death without prior relapse or at last follow-up.

### 2.6. Statistical Analysis

Gene expression data was analyzed using the NanoString nSolver^TM^ Advanced Analysis Software. Genes showing a fold change of >1.5 in the comparison of the *CD79B*-mutated vs. non-mutated group were considered biologically relevant. Further statistical tests were conducted with SPSS Statistics 25 (IBM, Armonk, NY, USA). The association of different parameters was tested using the two-sided Fisher’s exact test. The presence of a corresponding protein overexpression for the up-regulated genes was evaluated using the one-sided Fisher’s exact test. In the survival analysis (OS and RFS) using the Kaplan–Meier method, differences between categories were tested by the log rank test. Cox regression analysis was conducted to identify independent prognostic factors. Benjamini–Yekutieli correction for multiple testing was applied to control the false discovery rate (FDR), resulting in adjusted (adj.) *p*-values. The significance level was α = 0.05 overall. The tables and survival figures were generated using the R 4.3.1 software (R Foundation, Vienna, Austria) in RStudio (2025.05.1+513 ‘Mariposa Orchid’, Posit PBC, Boston, MA, USA).

## 3. Results

### 3.1. Clinical Information About the DLBCL Patient Population

The patient population comprised 48 primary, treatment-naïve DLBCLs (Table 1). *CD79B* ITAM alterations were detected in 35% (n = 17) of DLBCLs, which mostly comprised missense mutations affecting the first tyrosine of the ITAM domain (Y196) (Supplement Table S3). In total, 65% (n = 31) of cases did not harbor a *CD79B* mutation. No *CD79A* alterations were found. A *MYD88* L265P mutation was identified in 29% (n = 14) of patients. *CD79B* mutations frequently co-occurred with *MYD88* L265P, with 71% (n = 12) of *CD79B*-mutated lymphomas harboring a *MYD88* mutation (*p* < 0.05). Per Hans classifier, 54% (n = 26) of the lymphomas were categorized as non-GCB type and 42% (n = 20) as GCB type. *CD79B*-mutated DLBCLs were more prevalent of non-GCB type compared to GCB type—76% (n = 13) vs. 18% (n = 3). 65% (n = 31) of DLBCLs were diagnosed at an extranodal site, with 42% (n = 13) of these originating from the testicles. Notably, 59% (n = 10) of the specimens with *CD79B* mutations were localized in the testicles. Since p53 protein overexpression, which was detected in the *CD79B*-mutated group, is known to be associated with *TP53* mutation, we analyzed the mutational status of *TP53* in a secondary analysis [26]. In total, 25% (n = 12) of the DLBCLs showed a *TP53* mutation, which were mainly missense mutations (Supplement Table S4). We could not observe an association of *TP53* mutation with the *MYD88* and *CD79B* status, nor the GCB/non-GCB type.

### 3.2. Gene Expression Analysis with the PanCancer Pathways Panel

In total, 48 specimens characterized by mutational analysis for *CD79B* and *MYD88* were analyzed using the NanoString nCounter^®^ PanCancer Pathways Panel. Profiling of RNA expression revealed several up-regulated genes in *CD79B*-mutated vs. non-mutated cases (adj. *p* < 0.05), including 18 with a biologically relevant fold change > 1.5 (Table 2).

Downstream of CD79B in the BCR signaling pathway, *CARD11* was up-regulated, while *NF-κB* (*NFKB1*) RNA levels did not differ between the groups. An up-regulation of various direct NF-κB targets, such as *NFKBIZ*, *IL10*, *IL12A*, *PIM1* and the antiapoptotic *BCL2A1*, was detected in the *CD79B*-mutated group. Further up-regulated genes involved in apoptosis were *BID* and *granzyme B*. The differential expression of many genes not considered as NF-κB targets was also found in the *CD79B*-mutated group, with the overall strongest up-regulation for *ARNT2* and *WNT11* as well as genes involved in cell cycle regulation such as *RUNX1*, *RUNX1T1* and *RASGRF1*. Other up-regulated genes included *IL7*, *STAT3*, *MLLT4*, *CD14* and the *HSP90B1* subunit. *TP53* showed a significant differential expression, but its fold change did not reach the threshold of 1.5. In addition, 26 down-regulated genes were identified (Supplement Table S5).

### 3.3. Immunohistochemical Analysis

**Figure 1:**
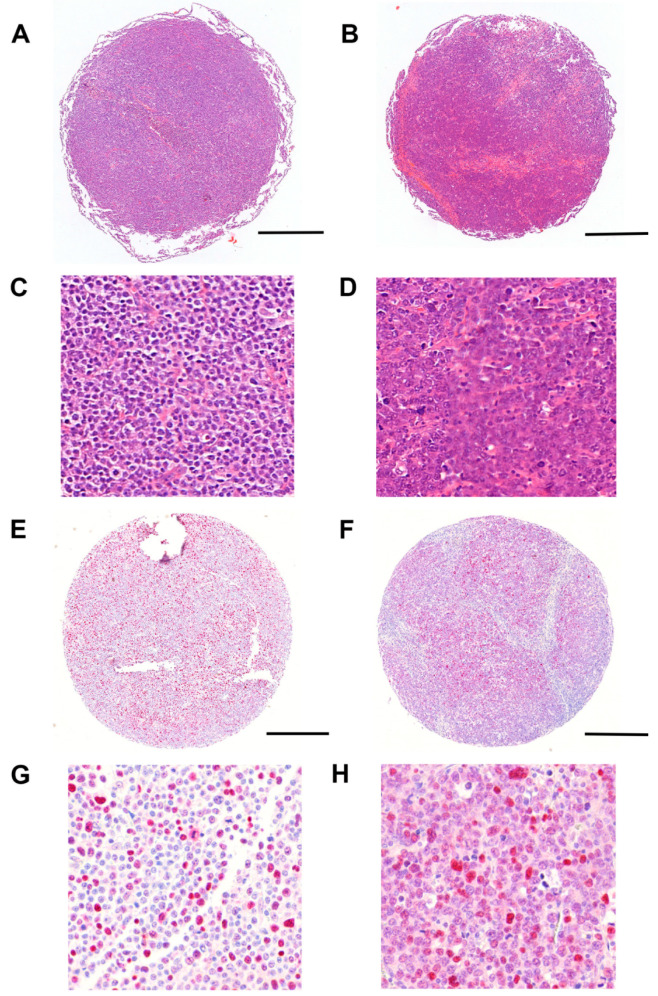
Representative H&E staining and p53 immunohistochemistry in *CD79B*-mutated and *CD79B* wild type (wt) DLBCLs. Low-magnification overviews of TMA cores are shown in (**A**) (wt, H&E), (**B**) (mutated, H&E), (**E**) (wt, p53 IHC) and (**F**) (mutated, p53 IHC). Corresponding high-magnification views are shown in (**C**) (for (**A**)); (**D**) (for (**B**)); (**G**) (for (**E**)) and (**H**) (for (**F**)). The cores demonstrated dense infiltrates of blastic lymphoma cells in both *CD79B* wild type (**A**,**C**) and mutated (**B**,**D**) DLBCLs. p53 protein expression was higher in *CD79B*-mutated cases (**F**,**H**) compared with wild type (**E**,**G**) (*p* < 0.05). This difference did not remain significant after correction for multiple testing. Scale bars: 500 µm (**A**,**B**,**E**,**F**; 5×) and 120 µm (**C**,**D**,**G**,**H**; 19.2×).

Immunohistochemical analysis was performed for a subset of the up-regulated genes identified in the RNA expression analysis to investigate whether corresponding protein overexpression could be detected. A significant difference at the protein level between the *CD79B*-mutated and wild type groups was observed only in the initial analysis for p53 (*p* < 0.05), which was weakly up-regulated at the RNA level (Figure 1). However, this difference did not remain significant after correction for multiple testing. Granzyme B was expressed independently of the *CD79B* mutation status in a non-neoplastic T-cell subpopulation rather than lymphoma cancer cells.

### 3.4. Survival Outcomes

**Figure 2:**
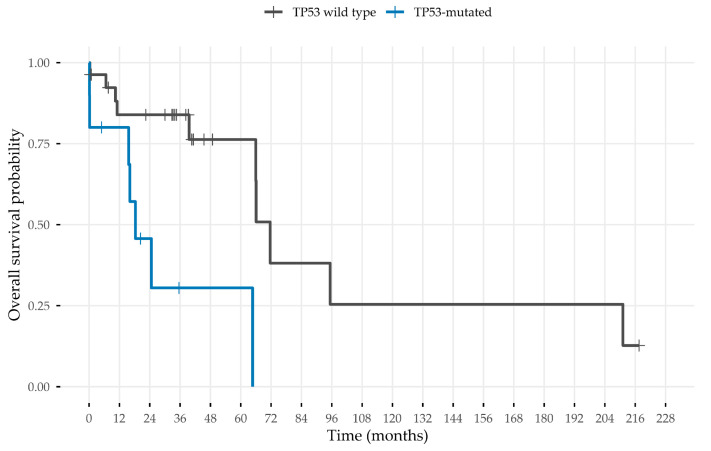
Survival analysis. Survival analysis revealed that *TP53* mutations were associated with a significantly worse overall survival (adj. *p* < 0.05).

The survival cohort comprised 85.4% (n = 41) of the overall 48 patients. A total of 14.6% (n = 7) were excluded due to missing survival data. Descriptive survival metrics are summarized in the supplement (Supplement Table S6). We investigated the associations of various clinical and molecular parameters related to RFS and OS using the log rank test. Clinical variables, the mutation status of *CD79B* and *MYD88*, as well as the detected up- and down-regulated genes were not associated with worse OS and RFS (Supplement Figure S1). Significantly shortened OS was observed for patients with a *TP53* mutation in a secondary analysis (adj. *p* < 0.05) (Figure 2). No independent prognostic factors were identified by Cox regression analysis.

## 4. Discussion

DLBCL is a non-Hodgkin’s lymphoma characterized by molecular heterogeneity [4,5,6]. DLBCLs can be subdivided into prognostic subgroups based on the cell of origin, the so-called GCB DLBCL, ABC DLBCL and unclassified cases [2,3,7]. The ABC subtype frequently harbors activating mutations in *CD79B* and *MYD88*, resulting in constitutive activation of the NF-κB pathway [10,11,12,13,14].

*CD79B* mutation was recently identified as an unfavorable prognostic factor for DLBCL patient survival and may be an important biomarker for DLBCL disease progression [25]. However, little is known about the gene and protein expression of DLBCLs with *CD79B* (and *MYD88*) mutations in comparison to their wild type counterparts. The current study compared the gene expression of 770 cancer-associated genes between a *CD79B*-mutated and a *CD79B* wild type primary, treatment-naïve DLBCL population. Most of the specimens with a *CD79B* mutation carried a concomitant *MYD88* mutation and were classified as non-GCB lymphomas, which is in concordance to previous studies [10,11]. The majority of *CD79B*-mutated lymphomas were localized in the testicles. Thus, this DLBCL group was characterized by a large proportion of entities from immunoprivileged sites. We already reported a high frequency of *CD79B* and *MYD88* mutations in testicular DLBCLs in a previous study [15].

RNA expression analysis detected, for several genes, a differential expression between *CD79B*-mutated and *CD79B* wild type cases, whereby we focused on the up-regulated genes. An up-regulation was found for *CARD11*, a downstream effector of *CD79B* in the BCR pathway, which is involved in the oncogenic activation of NF-κB in DLBCL [11,27]. Although the level of *NF-κB* (*NFKB1*) up-regulation in the *CD79B*-mutated group turned out to be minimal, we observed an altered gene expression of several direct NF-κB targets (*NFKBIZ*, *IL10*, *IL12A*, *PIM1* and *BCL2A1*) [28,29,30,31,32].

A strong up-regulation was observed for *NFKBIZ*, the gene encoding the atypical nuclear IκB family member IκBζ, which is indispensable for nuclear NF-κB activity in ABC DLBCLs and therefore for the survival of this DLBCL subtype [33]. The important role of the cytokine *IL10* in the survival and proliferation of ABC DLBCLs has already been described, in particular via *STAT3* signaling [34,35]. *STAT3* is a transcription factor, which is frequently constitutively activated in cancer as in ABC DLBCLs, involved in many central oncogenic processes and its gene was also up-regulated in the *CD79B*-mutated group [35,36,37]. Furthermore, it has been suggested that these three molecules form an autoregulatory feed-forward loop in ABC-DLBCLs, since all three proteins have been described as being overexpressed in this lymphoma subtype [33,35,37,38]. According to the literature, the constitutively active NF-κB pathway, as a result of a BCR signaling pathway mutation (e.g., *CD79B*), up-regulates IκBζ followed by the activation of a NF-κB target gene subset, including *IL10* [33,38]. Autocrine signaling mediated by this cytokine leads to the activation of STAT3, which in turn presumably promotes IκBζ expression, resulting in a protumorigenic effect of the malignant ABC DLBCL cells by sustaining the oncogenic NF-κB activity in the nucleus [35,38]. In addition to the concomitant up-regulation of the three genes involved in our study, further research is required to prove the existence and function of this feed-forward cycle or to investigate other roles and regulatory mechanisms of these genes in *CD79B*-mutated DLBCLs.

*PIM1* encodes a serine/threonine kinase that is known to be frequently mutated and overexpressed in DLBCL and plays an important protumorigenic role in this lymphoma [20,31,39,40]. *PIM1* was up-regulated in *CD79B*-mutated DLBCLs vs. *CD79B* wild type DLBCLs, indicating an important role of *PIM1* for *CD79B*-mutated DLBCLs. This is consistent with the characteristic mutations of the *PIM1* proto-oncogene together with *CD79B* and *MYD88* in the MCD subtype [21,23].

Our study also showed that the NF-κB target gene *BCL2A1*, an anti-apoptotic member of the BCL-2 family already known to be overexpressed in DLBCL, was up-regulated in *CD79B*-mutated DLBCLs [32,41,42]. BCL2A1 overexpression induces a protumorigenic and chemoresistant function in malignant cells and represents an attractive target in cancer [42].

We detected the up-regulation of further tumor-associated genes that are not NF-κB targets. The most strongly up-regulated gene in the *CD79B*-mutated group was *ARNT2*. ARNT2 is a transcription factor of the basic helix–loop–helix/Per-ARNT-SIM (bHLH/PAS) family, which is mainly expressed in the kidney and CNS [43,44]. The function and expression of ARNT2 in cancer seems to be tissue-dependent, and the underlying mechanisms are yet to be determined. One feature observed in hypoxic conditions, as found in the microenvironment of aggressive tumors, was the binding of the transcription factor to hypoxia-inducible factor (HIF)-1α in the nucleus, and this heterodimeric complex induced oncogenic processes in order to promote tumor growth [44,45,46]. A hypoxia-mediated activation leading to cancer progression was reported for *WNT11*, the second most up-regulated gene in the *CD79B*-mutated group [47]. This induction has been described as a result of the heterodimerization of HIF-1α with the ARNT2 homolog ARNT [47]. Although HIF-1α complexed with either ARNT or ARNT2 has been shown to promote similar gene expression profiles in hypoxic conditions, a corresponding mechanism of WNT11 activation via ARNT2 was not examined [46,47]. Neither *ARNT2* nor *WNT11* have, to our knowledge, been investigated in DLBCL so far. Given that they were the two most up-regulated genes in the *CD79B*-mutated DLBCL group in our study, further investigations are needed to determine whether they are involved in *CD79B*-mutated DLBCL development either separately or via a (HIF-1α)-ARNT2-mediated WNT11 activation and whether this feature can be used as a new therapeutic target in this lymphoma group.

*IL7*, *MLLT4*, *RASGRF1*, *HSP90B1*, *RUNX1T1* and *RUNX1* were up-regulated in *CD79B*-mutated DLBCLs. However, their potential involvement in DLBCL, to our knowledge, remains to be elucidated. As these genes were associated with other hematological and non-hematological cancers, further research is required to assess their oncogenic relevance in the *CD79B*-mutated group.

In survival analysis, *TP53* mutation was significantly associated with worse OS. This finding regarding the tumor suppressor gene is consistent with the results of several studies of DLBCL patients [26,48,49,50,51].

A limitation of the study is that the investigation of a corresponding protein overexpression for the up-regulated genes in the *CD79B*-mutated group was limited to immunohistochemical analysis of a small subset of the gene expression profile. Thus, only preliminary conclusions can be drawn about the protein expression profile in *CD79B*-mutated DLBCLs, requiring further studies in this regard.

## 5. Conclusions

In conclusion, the obtained gene expression profile of *CD79B*-mutated DLBCLs improves the characterization of this lymphoma subpopulation and contributes to a better understanding of the complex molecular heterogeneity of DLBCL. The results confirm the established characteristics of DLBCL and identify novel up-regulated genes in relation to *CD79B* mutation status. Further studies on independent, larger cohorts are required to investigate the relevance of these previously unrecognized genes in this context, potentially uncovering new therapeutic targets.

## Figures and Tables

**Table 1:** Clinical information about the 48 DLBCL patients and detailed information about the *CD79B*-mutated and wild type groups.

**Clinical Parameter Overall (n = 48)**		**Number (%)**
*CD79*		
* CD79B*-mutated		17 (35%)
* CD79A*-mutated		0 (0%)
Wild type		31 (65%)
*MYD88*		
Mutated (L265P)		14 (29%)
Wild type		32 (67%)
Not informative		2 (4%)
Maturation		
GCB type		20 (42%)
Non-GCB type		26 (54%)
Not informative		2 (4%)
Location		
Nodal		17 (35%)
Extranodal		31 (65%)
Testicular		13 (42%)
*TP53*		
Mutated		12 (25%)
Wild type		32 (67%)
Not informative		4 (8%)
**Clinical Parameter**	***CD79B*-Mutated** ** DLBCLs (n = 17)**	***CD79B* Wild Type** **DLBCLs (n = 31)**
	**Number (%)**	**Number (%)**
*MYD88*		
Mutated (L265P)	12 (71%)	2 (6%)
Wild type	4 (23%)	28 (90%)
Not informative	1 (6%)	1 (3%)
Maturation		
GCB type	3 (18%)	17 (55%)
Non-GCB type	13 (76%)	13 (42%)
Not informative	1 (6%)	1 (3%)
Location		
Testicular	10 (59%)	3 (10%)
Non-testicular	7 (41%)	28 (90%)

**Table 2:** Up-regulated genes in *CD79B*-mutated DLBCLs compared to *CD79B* wild type DLBCLs.

Gene	Description	Fold Change	Adj. *p*-Value
*ARNT2*	aryl-hydrocarbon receptor nuclear translocator 2	4.78	0.0001
*WNT11*	wingless-type MMTV integration site family, member 11	3.04	0.0072
*GZMB*	granzyme B (granzyme 2, cytotoxic T-lymphocyte-associated serine esterase 1)	2.89	0.0046
*IL10*	interleukin 10	2.70	0.0024
*IL12A*	interleukin 12A (natural killer cell stimulatory factor 1, cytotoxic lymphocyte maturation factor 1)	2.62	0.0003
*NFKBIZ*	nuclear factor of kappa light polypeptide gene enhancer in B-cells inhibitor, zeta	2.61	0.0001
*RASGRF1*	Ras protein-specific guanine nucleotide-releasing factor 1	2.48	0.0011
*IL7*	interleukin 7	2.15	0.0001
*PIM1*	pim-1 oncogene	2.11	0.0002
*RUNX1T1*	runt-related transcription factor 1; translocated to, 1 (cyclin D-related)	2.00	0.0406
*CARD11*	caspase recruitment domain family, member 11	1.96	0.0034
*BCL2A1*	BCL2-related protein A1	1.77	0.0307
*STAT3*	signal transducer and activator of transcription 3 (acute-phase response factor)	1.76	0.0001
*HSP90B1*	heat shock protein 90 kDa beta (Grp94), member 1	1.76	0.003
*MLLT4*	myeloid/lymphoid or mixed-lineage leukemia (trithorax homolog, Drosophila); translocated to, 4	1.62	0.0032
*RUNX1*	runt-related transcription factor 1	1.59	0.0005
*CD14*	CD14 molecule	1.57	0.034
*BID*	BH3 interacting domain death agonist	1.54	0.0054
*TP53*	tumor protein p53	1.37	0.022
*NFKB1*	nuclear factor of kappa light polypeptide gene enhancer in B-cells 1	1.25	0.0352

## Data Availability

The datasets generated and analyzed during the current study are available from the corresponding author on reasonable request.

## Supplementary Materials

The following supporting information can be downloaded at https://www.mdpi.com/article/10.3390/jpm15110548/s1: Table S1: Clinical information about the patient population; Table S2: Primary antibodies used in this study for immunohistochemistry; Table S3: Detected mutations in the *CD79B* gene; Table S4: Detected mutations in the *TP53* gene; Table S5: Down-regulated genes in *CD79B*-mutated DLBCL compared to *CD79B* wild type DLBCL; Table S6: Descriptive survival metrics; Figure S1: Survival analysis. Kaplan–Meier curves for *CD79B* and *MYD88* mutation.
