# Supplementary material for: Gene Expression Profiling Provides an Improved Characterization of CD79B-Mutated Diffuse Large B-Cell Lymphomas
Source: J Pers Med. 2025 Nov 10;15(11):548. doi: 10.3390/jpm15110548 (PMC12653173; doi:10.3390/jpm15110548)

Table S1. Clinical information about the patient population. Abbreviations: R, rituximab; C, cyclophosphamide; H, doxorubicin hydrochloride; O, vincristine sulfate; P, prednisone.

**Clinical parameter overall (n = 48)**	
**Gender**	Number (%)
Female	16 (33%)
Male	32 (67%)
**Age at diagnosis**	Years
Years range	50 - 93
Mean ± SD	71.3 ± 11.0
Median	71.0
Female mean ± SD	71.1 ± 13.6
Female median	74.0
Male mean ± SD	71.4 ± 9.8
Male median	71.0
**Therapy (first-line)**	Number (%)
R-CHOP	6 (12.5%)
R-CHOP + Radiation	5 (10.4%)
R-CHOP + Surgery	3 (6.3%)
R-CHOP + Bendamustine	1 (2.1%)
R-CHOP + Radiation + Surgery	2 (4.2%)
R-CHOP + Radiation + Bendamustine	1 (2.1%)
R-CHOP + Surgery + Bendamustine	1 (2.1%)
CHOP	2 (4.2%)
CHOP + Radiation	1 (2.1%)
CHOP + Surgery	4 (8.3%)
Radiation	2 (4.2%)
Surgery	2 (4.2%)
Radiation + Surgery	1 (2.1%)
No therapy	17 (35.4%)

Table S2. Primary antibodies used in this study for immunohistochemistry.

Antibody	Antigen (Clone)	Provider	Dilution	Epitope Retrieval	Incubation
Mouse IgG1	BCL-6 (SP155)	Zytomed Systems GmbH, Germany	1:10	Pressure cooker in citrate buffer 30min	1 hour at RT
Mouse IgG1	CD10 (56C6)	Zytomed Systems GmbH, Germany	1:40	Pressure cooker in citrate buffer 30min	1 hour at RT
Rabbit IgG	Cyclin D1 (SP4)	Zytomed Systems GmbH, Germany	1:50	Pressure cooker in citrate buffer 30min	1 hour at RT
Rabbit IgG	CD3 (SP7)	Zytomed Systems GmbH, Germany	1:100	Pressure cooker in citrate buffer 30min	1 hour at RT
Mouse IgG2ak	CD20 (L26)	Zytomed Systems GmbH, Germany	1:100	No pretreatment	1 hour at RT
Mouse IgG1k	CD79a (JCB117)	Agilent Technologies Inc., USA	1:100	Pressure cooker in citrate buffer 20min	1 hour at RT
Mouse IgG1k	MUM-1 (MUM-1 p)	Agilent Technologies Inc., USA	1:100	Pressure cooker in citrate buffer 20min	1 hour at RT
Rabbit IgG	CD23 (SP163)	Zytomed Systems GmbH, Germany	1:100	Pressure cooker in citrate buffer 20min	1 hour at RT
Mouse IgG1k	BCL-2 (124)	Agilent Technologies Inc., USA	1:200	Pressure cooker in citrate buffer 20min	1 hour at RT
Mouse IgG1	Ki-67 (K-2)	Zytomed Systems GmbH, Germany	1:500	Pressure cooker in citrate buffer 20min	1 hour at RT
Mouse IgG2a	P53 (BP53-12)	Zytomed Systems GmbH, Germany	1:50	Pressure cooker in citrate buffer 20min	1 hour at RT
Rabbit IgG	PIM1 (D8D7Y)	Cell Signaling Technology, USA	1:200	Pressure cooker in citrate buffer 20min	1 hour at RT
Rabbit IgG	HSP90 (C45G5)	Cell Signaling Technology, USA	1:250	Pressure cooker in citrate buffer 20min	1 hour at RT
Mouse IgG2a	STAT3 (124H6)	Cell Signaling Technology, USA	1:500	Pressure cooker in citrate buffer 20min	1 hour at RT
Rabbit IgG	NF-κB p65 (D14E12)	Cell Signaling Technology, USA	1:500	Pressure cooker in citrate buffer 20min	1 hour at RT
Mouse IgG1k	IL7 (D-9)	Santa Cruz Biotechnology Inc., USA	1:100	Pressure cooker in citrate buffer 20min	1 hour at RT
Goat polyclonal IgG	IL10 (M-18)	Santa Cruz Biotechnology Inc., USA	1:100	Pressure cooker in citrate buffer 20min	1 hour at RT

Antibody	Antigen (Clone)	Provider	Dilution	Epitope Retrieval	Incubation
Mouse IgG2ak	Granzyme B (2C5)	Santa Cruz Biotechnology Inc., USA	1:100	Pressure cooker in citrate buffer 20min	1 hour at RT

Table S3. Detected mutations in the *CD79B* gene.

Gene level	Protein level	Quantity
c.587A>G	p.Y196C	8
c.586T>C	p.Y196H	3
c.586T>G	p.Y196D	1
c.587A>T	p.Y196F	1
c.592_594dup	p.G198dup	1
c.574G>T	p.E192*	1
c.576_586del	p.D193Rfs*?	1
c.611dupC	p.A205Sfs*?	1

Table S4. Detected mutations in the *TP53* gene.

**Gene level**	**Protein level**
c.386C>T	p.A129V
c.394A>G	p.K132E
c.430C>T	p.Q144*
c.452C>A	p.P151H
c.467G>A	p.R156H
c.487T>C	p.Y163H
c.571C>T	p.P191S
c.574C>T	p.Q192*
c.700T>C	p.Y234H
c.770T>C	p.L257P
c.838A>G	p.R280G
c.220del	p.A74Pfs*?
c.445del	p.S149Pfs*?
c.847delinsTGGATGT	p.R283delinsWMC

Table S5. Down-regulated genes in *CD79B* mutated DLBCL compared to *CD79B* wild type DLBCL.

Gene	Description	Fold change	Adj. p-value
BMP7	*bone morphogenetic protein 7*	-3.37	0.0194
CDH1	*cadherin 1, type 1, E-cadherin (epithelial)*	-2.94	0.0012
MAPK10	*mitogen-activated protein kinase 10*	-2.65	0.0124
IL22RA2	*interleukin 22 receptor, alpha 2*	-2.47	0.0104
PRKAR2B	*protein kinase, cAMP-dependent, regulatory, type II, beta*	-2.36	0.0039
CDKN2A	*cyclin-dependent kinase inhibitor 2A*	-2.17	0.0008
LAMC2	*laminin, gamma 3*	-2.17	0.0015
MMP7	*matrix metallopeptidase 7 (matrilysin, uterine)*	-2.17	0.0119
WNT5B	*wingless-type MMTV integration site family, member 5B*	-2.13	0.0091
PIK3CG	*phosphatidylinositol-4,5-bisphosphate 3-kinase, catalytic subunit gamma*	-1.92	0.0022
DDIT4	*DNA-damage-inducible transcript 4*	-1.84	0.0031
EFNA5	*ephrin-A5*	-1.82	0.0068
CDKN2B	*cyclin-dependent kinase inhibitor 2B (p15, inhibits CDK4)*	-1.79	0.0438
TCF3	*transcription factor 3*	-1.69	0.0041
FGF14	*fibroblast growth factor 14*	-1.67	0.0322
IL1R2	*interleukin 1 receptor, type II*	-1.66	0.035
FGFR4	*fibroblast growth factor receptor 4*	-1.64	0.0256
CDKN2C	*cyclin-dependent kinase inhibitor 2C (p18, inhibits CDK4)*	-1.63	0.0152
RRAS2	*related RAS viral (r-ras) oncogene homolog 2*	-1.57	0.0316
BMPR1B	*bone morphogenetic protein receptor, type IB*	-1.57	0.0455
FGFR1	*fibroblast growth factor receptor 1*	-1.56	0.0211
TIAM1	*T-cell lymphoma invasion and metastasis 1*	-1.55	0.0377
DDIT3	*DNA-damage-inducible transcript 3*	-1.54	0.0032
EGFR	*epidermal growth factor receptor*	-1.54	0.0229
CCND1	*cyclin D1*	-1.54	0.0308
FGF22	*fibroblast growth factor 22*	-1.51	0.0496

Table S6. Descriptive survival metrics.

**Survival analysis (n = 41)**	
**Relapse free survival (RFS)**	Number (%)
Censored	30 (62.5%)
Relapse of disease	11 (22.9%)
Unknown	7 (14.6%)
	**Months**
Mean follow-up time ± SD	35.8 ± 44.5
Median follow-up (reverse Kaplan-Meier) [IQR]	33.9 [15.6–45.5]
Range	0.2 - 211.2
**Overall survival (OS)**	Number (%)
Censored	22 (45.8%)
Death	19 (39.6%)
Unknown	7 (14.6%)
	**Months**
Mean follow-up time ± SD	40.7 ± 48.2
Median follow-up (reverse Kaplan-Meier) [IQR]	39.3 [32.8–217.5]
Range	0.2 - 217.5

Figure S1. Survival analysis. Kaplan-Meier curves for *CD79B* and *MYD88* mutation.

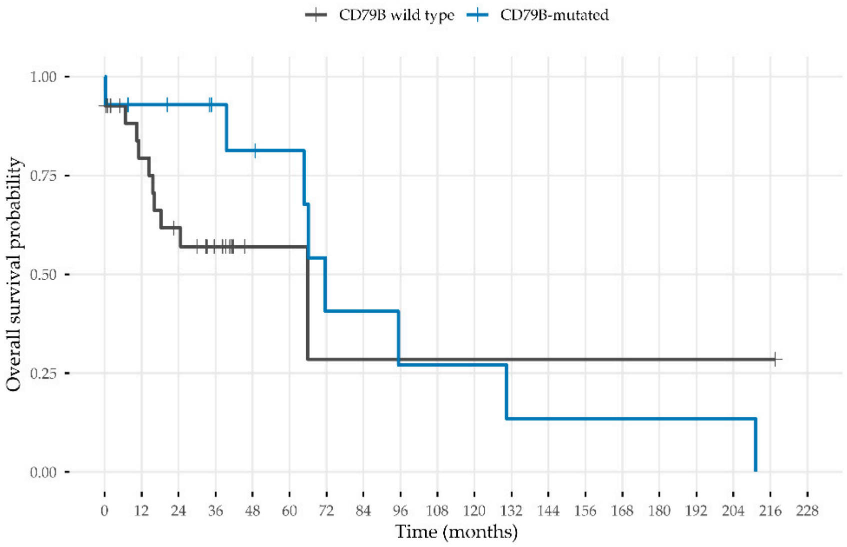

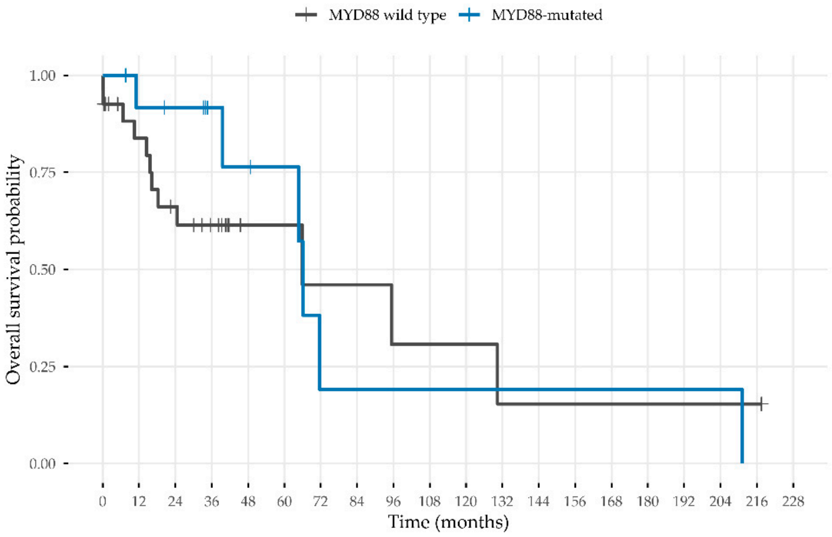

Supplement: Supplementary file 1 [file jpm-15-00548-s001.zip › jpm-3837571-supplementary.pdf]
